# Intention to adopt clinical decision support systems in a developing country: effect of Physician’s perceived professional autonomy, involvement and belief: a cross-sectional study

**DOI:** 10.1186/1472-6947-12-142

**Published:** 2012-12-05

**Authors:** Murali Sambasivan, Pouyan Esmaeilzadeh, Naresh Kumar, Hossein Nezakati

**Affiliations:** 1 Global Entrepreneurship Research and Innovation Center (GERIC), Universiti Malaysia Kelantan, Kelantan, Malaysia; 2 Graduate School of Management, Universiti Putra Malaysia, Putra, Malaysia; 3 Global Entrepreneurship Research and Innovation Center (GERIC), Universiti Malaysia Kelantan, Kelantan, Malaysia; 4 Faculty of Economics and Management, Universiti Putra Malaysia, Putra, Malaysia

**Keywords:** Clinical decision support system, Professional autonomy, Performance expectancy, Effort expectancy,Participation in decision making, Intention to use, Physicians, Malaysia

## Abstract

**Background:**

Computer-based clinical decision support systems (CDSS) are regarded as a key element to enhance decision-making in a healthcare environment to improve the quality of medical care delivery. The concern of having new CDSS unused is still one of the biggest issues in developing countries for the developers and implementers of clinical IT systems. The main objectives of this study are to determine whether (1) the physician’s perceived professional autonomy, (2) involvement in the decision to implement CDSS and (3) the belief that CDSS will improve job performance increase the intention to adopt CDSS. Four hypotheses were formulated and tested.

**Methods:**

A questionnaire-based survey conducted between July 2010 and December 2010. The study was conducted in seven public and five private hospitals in Kuala Lumpur, Malaysia. Before contacting the hospitals, necessary permission was obtained from the Ministry of Health, Malaysia and the questionnaire was vetted by the ethics committee of the ministry. Physicians working in 12 hospitals from 10 different specialties participated in the study. The sampling method used was stratified random sampling and the physicians were stratified based on the specialty. A total of 450 physicians were selected using a random number generator. Each of these physicians was given a questionnaire and out of 450 questionnaires, 335 (response rate – 74%) were returned and 309 (69%) were deemed usable.

**Results:**

The hypotheses were tested using Structural Equation Modeling (SEM). Salient results are: (1) Physicians’ perceived threat to professional autonomy lowers the intention to use CDSS (p < 0.01); (2) Physicians involvement in the planning, design and implementation increases their intention to use CDSS (p < 0.01); (3) Physicians belief that the new CDSS will improve his/her job performance increases their intention to use CDSS (p < 0.01).

**Conclusion:**

The proposed model with the three main constructs (physician’s professional characteristic, involvement and belief) explains 47% of the variance in the intention to use CDSS. This is significantly higher than the models addressed so far. The results will have a major impact in implementing CDSS in developing countries.

## Background

A clinical decision support system (CDSS) is regarded as an application of decision support system (DSS) which takes patient data as input and generates patient-specific advice. These knowledge-based systems through a process of reasoning techniques generate diagnostic and treatment options and care planning [1,2]. The use of IT in health care practices is mainly for two purposes: creating and maintaining electronic medical record (EMR) of each patient and integrating those records using computerized decision support systems to generate specific medical advice [3]. CDSS applications in healthcare are regarded as a key element to enhance decision-making in a healthcare environment to improve the quality of medical care delivery and many CDSSs have been shown to improve physician performance [4]. CDSS uses include: alerts and reminders, diagnostic assistance, therapy critiquing and planning, prescribing decision support, information retrieval and image recognition and interpretation [5]. However, factors affecting the physicians’ IT adoption behavior are not completely clear [6,7]. The concern of having new CDSS unused is still one of the biggest issues for the developers and implementers of clinical IT systems [8]. Besides low usage, problems in the information systems for patient care can also occur while (1) entering and retrieving information and (2) communicating and coordinating the information for decision-making [9]. Therefore, proper management of CDSS and related systems are critical for providing proper healthcare.

Growing use of IT in healthcare in developed countries has been driven by the belief and the evidence that these systems can help enhance the quality of health care [10]. A recent study involving outpatient physicians in US shows that EMR and CDSS are being used in 30% and 17% of the patient visits, respectively [11]. The health care systems and use of IT in developed countries have been in existence for at least two decades more than the developing countries and these countries pose a much greater challenge in implementing computerized decision support systems [12]. There are many critical reasons cited for the problems of low usage of CDSS in developing countries: (1) dependence on EMR to supply the relevant data and the problems in implementing EMR, (2) poor human interface design, (3) problems in fitting CDSS into the routine process of patient care, (4) reluctance of physicians to use the system, (5) computer illiteracy of physicians and (6) cost of purchase and implementation [5].

A popular framework that has been developed and used extensively to study the usage behavior of IT systems is the Unified Theory of Acceptance and Use of Technology (UTAUT) model [13]. However, this model which unifies eight different models is for general applications and does not take into account the unique characteristics of the users. A recent study on usage of EMR based on UTAUT shows that the model can explain only 20% of the variance in the usage intention of EMR [14]. In our study, we propose other factors that take into account the characteristics of physicians that play a critical role in the acceptance and usage of technology [15] such as CDSS especially, in developing countries.

The aim of our research work for this paper is to determine the factors that influence adoption and therefore, use of clinical decision support systems by physicians in hospitals. In this research, we do not address private clinical practices. Specifically, we study the effect of physician’s perceived threat to professional autonomy [7], physician’s level of involvement in deciding the implementation of CDSS [16], the physician’s belief that the new CDSS will improve his/her job performance (Performance expectancy) [13] and the degree of ease associated with the use of the system (Effort expectancy) [13].

In order to develop a framework for this study, we used UTAUT model as a base. However, we did not consider the constructs, facilitating conditions and subjective norms used in the original UTAUT model. When the constructs such as performance expectancy and effort expectancy come into play, facilitating conditions become insignificant in explaining and in predicting intention especially in a pre-implementation study [13]. Moreover, the empirical results signify that facilitating conditions have a direct effect on actual usage and not on behavioral intention [12]. There are studies that indicate the insignificant role of subjective norms in healthcare professional’s decision making about using IT because of the self-autonomy of the professionals [1,15,17,18]. There are studies that report otherwise [19,20]. However, in this research we consider only the unique characteristics of healthcare professionals. Due to these reasons, we omitted facilitating conditions and subjective norms.

## Methods

### Study site and sample

The study was conducted in Malaysia, a fast developing country in South-East Asia. Physicians from seven public and five private hospitals participated in this study. The hospitals are located in and around the capital city, Kuala Lumpur. An approval letter to conduct the study was obtained from the Ministry of Health after the ethical committee vetted and approved the questionnaire and the study plan. This letter helped us gain access to the physicians in these hospitals. The sampling method used was stratified random sampling and the physicians were stratified based on the specialty. The size of the hospital (in terms of total number of physicians) was used to determine the number of physicians to be sampled from a particular hospital. At the next level, within each hospital the number of physicians sampled in each specialty was determined by the ratio of physicians in that specialty (department) to the total number of physicians in that hospital. The sample size was calculated using the sample size calculator available online. The calculator recommended a sample size of 378 and we used a larger sample size. A total of 450 physicians were selected using a random number generator. Each of these physicians was given a questionnaire and out of 450 questionnaires, 335 (response rate – 74%) were returned and 309 (69%) were deemed usable. Twenty six questionnaires that were excluded from further analysis had 30% or more of the questions (items) unanswered. The study was conducted between July 2010 and December 2010.

### Measures

The questionnaire constructed for the study consisted of five constructs and all the constructs were measured using a 5-point Likert scale (5 – strongly agree; 1 – strongly disagree). The constructs were taken from established sources: Intention to use CDSS [15] (six items), Perceived threat to professional autonomy [7] (six items), Level of physician’s involvement in decision-making [16] (four items), Performance expectancy [13] (six items) and Effort expectancy [13] (six items). Among these constructs, intention to use CDSS had two items and effort expectancy one item that were negatively worded. These items were reverse coded for further analysis. The questionnaire was constructed in English. Since the professionals in Malaysia were adept at handling English language, there was no need to translate the questionnaire to the national language of Malaysia (Bahasa Malaysia). The construct validity of the five constructs was assessed using Confirmatory Factor Analysis (CFA).

### Hypotheses development

**Figure 1:**
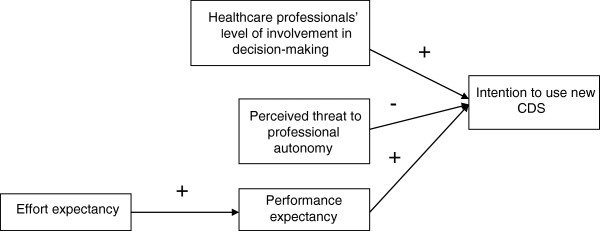
Theoretical Framework.

The conceptual framework used in this study is given in Figure 1. Professional autonomy is viewed as a precious privilege given to physicians and they do not like to lose it in their workplace. Physicians maintain factors that protect their professional autonomy and react negatively to the elements that may invalidate their professional autonomy and traditional work practice. Perceived threat to professional autonomy is defined as the degree to which a physician believes that using a particular system decreases his/her control over the conditions, processes, or contents of his/her work [7]. This study hypothesizes that perceived threat to professional autonomy reduces physician’s intention to use CDSS. Based on the above arguments, we hypothesize as follows:

Hypothesis1: There is a negative relationship between physicians’ perceived threat to professional autonomy and their intention to use the CDSS system.

The significant role of performance expectancy among physicians in shaping their intention toward using a new technology is centered on physicians’ utility-based point of view about using the technology [17]. Performance expectancy exerts the most significant impact on physicians’ intention to use CDSS [1]. The proposed framework hypothesizes that performance expectancy helps physicians in forming their intention to use the CDSS and is as follows:

Hypothesis2: There is a positive relationship between physicians’ performance expectancy and their intention to use the CDSS system.

Some studies have found that effort expectancy does not directly affect users’ behavioral intention to use the system and its effect can be enhanced through performance expectancy [13]. This study hypothesizes that effort expectancy positively affects performance expectancy in accepting CDSS among healthcare professionals [21] and is as follows:

Hypothesis3: There is a positive relationship between effort expectancy and performance expectancy in using CDSS among physicians.

The literature states that if physicians are involved in decision-making process regarding the introduction and development of appropriate IT system in organizations, they become more willing to change their long-term practice pattern [22] and this results in lower costs and improved healthcare delivery [23]. Physician’s high level of involvement in decision making regarding the development and implementation of CDSS can positively influence the intention to use CDSS [23]. Based on the above arguments, we hypothesize as follows:

Hypothesis4: Physician’s level of involvement in making decisions regarding the development and implementation of CDSS is positively related to the intention to use CDSS.

### Analysis

A two-step data analysis approach (measurement model and structural model) of the structural equation model was applied. A confirmatory factor analysis (CFA) was performed to verify the construct validity of each construct [24]. Two items from each construct were removed to achieve a better fit. Construct reliability was assessed using evaluation of the composite reliability, Average Variance Extracted (AVE) and Cronbach’s alpha value. All constructs exhibited composite reliability and Cronbach’s alpha greater than the acceptable level of 0.7 indicating that the measurement errors were relatively small [25]. To assess the discriminant validity between constructs, the test that requires AVE for each construct to be higher than the squared correlation between the two associated latent variables was performed. All factors met the criteria for discriminant validity as shown in Table 1[25].

**Table 1:** Cronbach’s Alpha (CR) and Composite reliability (COMP) of constructs (diagonal of the matrix contains the Average Variance Extracted (AVE) and off-diagonal elements are the squared correlations between constructs)

**Constructs**	**CR**	**COMP**	**INTENTION**	**INVOLVEMENT**	**PERCEIVED THREAT**	**EFFORT EXPECTANCY**	**PERFORMANCE EXPECTANCY**
INTENTION	0.850	0.830	0.630				
INVOLVEMENT	0.913	0.870	0.162	0.640			
PERCEIVED THREAT	0.890	0.890	0.223	0.016	0.670		
EFFORT EXPECTANCY	0.900	0.930	0.274	0.108	0.038	0.700	
PERFORMANCE EXPECTANCY	0.920	0.930	0.209	0.108	0.019	0.489	0.700

**Figure 2:**
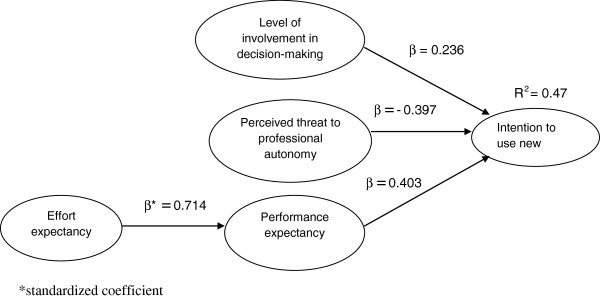
Structural model results.

After confirming the measurement model, the structural model was then examined. The model fit indices are: Comparative Fit Index – 0.91, Incremental Fit Index – 0.91, Tucker Lewis Index – 0.90, Root Mean Square Residual = 0.057, Root Mean Square Error of Approximation – 0.052, Normed Chi-Square – 1.833. These indices are within the prescribed limits and therefore, the model reflects a good fit to the data [26]. The model results are indicated in Figure 2.

## Results and discussion

### Respondent characteristics

A total of 450 physicians across 10 different specialties and 12 hospitals were targeted for the survey, 335 physicians participated and responses from 309 physicians were used for further analysis. Table 2 lists the respondents’ demographic characteristics. Approximately equal numbers of men and women were represented. Sixty three percent of the physicians had experience between six and 20 years. Seventy two percent of the physicians reported moderate to very high level of familiarity with clinical IT and about 90% reported little or no experience with CDSS. Sixty six percent of the physicians were from public hospitals. In developing countries, majority of the population get their medical treatment in public hospitals because of their affordability. Demographic information has not been used for further analysis. Earlier studies on adoption of EMR and Telemedicine have shown that effects of demography are overshadowed by professional characteristics of physicians [8,14].

**Table 2:** Demographic Characteristics of Respondents

**Characteristic**	**Category**	**Frequency**	**Percent**
Gender	Male	144	46.6
	Female	156	53.4
Age	20-29	36	11.7
	30-39	161	52.1
	40-49	75	24.3
	50-59	26	8.4
	60-69	10	3.2
	Over 70	1	0.3
Working Experience	1-5	74	23.0
	6-10	95	30.0
	11-20	102	33.0
	21-30	25	9.0
	Over 30	13	5.0
Specialty Areas	Anesthesiologist	27	8.7
	Geriatric	21	6.8
	Gen Prac	45	14.6
	Gynecologist	34	11.0
	Internist	33	10.7
	Pathologist	24	7.8
	Pediatric	39	12.6
	Psychiatrist	19	6.1
	Radiologist	22	7.1
	Surgeon	45	14.6
Level of familiarity with Clinical IT	Very low	16	5.2
	Low	70	22.7
	Moderate	173	56.0
	High	46	14.8
	Very High	4	1.3
Past experience in using CDSS	High	32	10.4
	Little/No	277	89.6
Type of Hospital	Public	204	66.0
	Private	105	34.0

### Descriptive statistics

Table 3 lists the descriptive scores of all the constructs and the correlation coefficients between the constructs. The mean scores indicate the following: (1) Physicians in Malaysia have a ‘moderate’ intention to use CDSS; (2) Hospital managers in Malaysia involve physicians to a ‘lesser extent’ while deciding to implement CDSS inhospitals; (3) Physicians in Malaysia perceive ‘some degree’ of threat from CDSS; (4) Physicians in Malaysia perceive that degree of ease associated with the use of CDSS is ‘not high’; and (5) Physicians belief that CDSS will improve his/her job performance is ‘high’.

**Table 3:** Descriptive statistics of constructs

**Construct**	**Mean**	**SD**	**Intention**	**Involvement**	**Perceived threat**	**EE**	**PE**
			**Correlation**				
INT	3.5129	0.82439	1.00	.403*	-.472*	.523*	.457*
INV	2.8786	0.99728		1.00	-.125	.328*	.328*
THREAT	3.1489	0.91062			1.00	-.139	-.196
EE	3.1126	0.79968				1.00	.699*
PE	3.7238	0.66066					1.00

*significant at 0.01 significance level.

Legend: EE – Effort Expectancy, PE – Performance Expectancy.

### Structural model results

The hypotheses were tested based on the structural model and the results are:

1) There is a significant negative relationship between perceived threat to professional autonomy and intention to use CDSS (*r (standardized coefficient) = −0.397, p-value = 0.00 < 0.05*).

2) There is a significant positive relationship between level of involvement in decision making and intention to use new CDSS (*r = 0.236, p-value= 0.00 < 0.05*).

3) There is a significant positive relationship between performance expectancy and intention to use new CDSS (*r = 0.403, p-value= 0.00 < 0.05*).

4) There is a significant positive relationship between effort expectancy and performance expectancy (*r = 0.714, p-value= 0.00 < 0.05*).

Level of physicians’ involvement in decision making, performance expectancy and perceived threat to autonomy collectively explain 47% of the variance in intention to use CDSS among physicians in Malaysia.

## Discussion

This survey on physicians’ intention to use CDSS was undertaken on the premise that physicians are different from other IT users and popular models such as UTAUT cannot be used to predict physicians’ acceptance behavior. A physician’s decision to accept a decision support system depends upon the following factors: perceived threat to professional autonomy, level of involvement in deciding the implementation of CDSS and belief that new CDSS will improve performance. Our research reveals that physicians IT adoption behavior depends on whether or not a CDSS threatens their professional autonomy. Why should a physician feel threatened? Literature suggests several reasons for this behavior. First, the physician’s belief that the new CDSS may erode the natural flow of his/her work routines and may not follow his/her practice patterns [7]. Second, the concern that his/her knowledge may be organized, codified and distributed to peers and other non-professionals [27]. Third, physicians feel uncomfortable when they face regulations and instructions generated by a CDSS that advises them on what to do [28]. Fourth, a misconception among physicians in Malaysia is that a CDSS can replace them [29]. Consequently, the possibility of using CDSS by physicians decreases. In a developing country like Malaysia, there is a strong need for continued motivation and training for physicians for the success of CDSS implementation initiatives.

The finding that performance expectancy is positively and significantly related to intention to use new CDSS is in line with previous studies that claim performance expectancy as an important determinant of physicians’ intention to use a new technology [1,30]. A plausible reason for a large effect of performance expectancy is rooted in the characteristics of respondents who are considered as pre-adopters. As indicated earlier, physicians in Malaysia can be considered as pre-adopters since 90% have little or no experience in using CDSS. This group focuses mainly on usefulness of CDSS and instrumental benefits that can be derived when forming their intentions to use a clinical IT [13]. As a result, performance expectancy is positively related to physicians’ intention to use CDSS.

The results concerning the effect of effort expectancy are consistent with some research on the behavior of physicians. For instance, some studies present a positive and significant relationship between effort and performance expectancies in the healthcare context. These studies signify the role of effort expectancy to use a new IT system in obtaining more utility from the system [31]. Based on this point of view, effort expectancy is considered as an important factor influencing physicians indirectly through performance expectancy. In light of the salient role of performance expectancy, this study shows that if physicians find the CDSS easy to use, they expect the system to be useful in increasing their productivity. Therefore, effort expectancy is found to be an antecedent for performance expectancy.

Literature states the importance of giving managerial roles to physicians [32]. Involvement of physicians in decision-making process regarding a new CDSS includes: participation in the planning, involvement in the implementation and participation in the development. When the level of involvement for physicians notably increases, they perceive that they are a part of the process that influences implementation of a new CDSS. Under such conditions, physicians perceive themselves as active stakeholders and they become more willing to change their traditional work routine by using CDSS [32].

What are the lessons learnt? Implementing and using CDSS in a developing country like Malaysia can be a challenging task. A suitable electronic infrastructure is vital to the use of CDSS [32]. Our results suggest that the hospital administrators must take following initiatives before implementing CDSS. First, the managers should realize a strong need for continued motivation and training for physicians [30]. The training and motivation can be provided by sending physicians for short-term attachments to hospitals (local or overseas) that have successfully implemented CDSS and by sending them to relevant conferences and seminars. Second, the new CDSS must have easy features and include user-friendly elements for the physicians to perceive that using the instructions given by the system is easy and will help them attain gains in job performance. Difficulty in processing complex features of CDSS makes physicians believe that the system is not useful and is not fit for their job. The hospital management along with the physicians must study different systems before deciding the right CDSS. Third, this study recommends that hospitals’ top managers and shareholders have to pay more attention to physicians’ participation in planning, development and implementation of a new CDSS. This study suggests that physicians be made to actively participate in the decision making process. If the physicians see that they are involved in general decision making about the CDSS, the possibility of showing negative reaction becomes lower. A recent study has identified ten different themes that need attention if a clinic or community hospital plans to implement and utilize CDSS [4]. Through our study, we propose three more themes that are relevant in hospitals in developing countries: physician’s characteristic (autonomy), physician’s involvement in developing CDSS and physician’s belief about CDSS.

## Conclusions

From a theoretical standpoint and theory building, the research contributes to IT adoption theories explaining user’s intention to accept new technology. Our research has attempted to identify important constructs from the user acceptance literature by using the popular UTAUT as a base model. Since the UTAUT is general and cannot address physicians’ unique characteristics, this model has been improved to better explain physicians’ IT adoption behavior in a hospital setting. Our model can explain 47% of the variance in physicians’ CDSS adoption behavior. The key findings of this study are: (1) Physicians’ perceived threat to professional autonomy lowers the intention to use CDSS; (2) Physicians involvement in the planning, design and implementation increases their intention to use CDSS; and (3) Physicians belief that the new CDSS will improve his/her job performance increases their intention to use CDSS. Our study not only identified the source of resistance but also suggested strategies to improve physicians’ behavior towards CDSS acceptance. Only with greater acceptance by physicians, new technology can play a significant role in advancing health care delivery. The level of resistance to use CDSS is higher in developing countries when compared to developed countries. The administrators of public and private hospitals must understand the factors that affect CDSS adoption and must take proactive steps before implementation.

Our study had several limitations. First, the study included private and public hospitals in Malaysia. The factors influencing the implementation of CDSS in hospitals could be different in other developing countries [28]. Second, our study considered the hospitals in and around the capital city, Kuala Lumpur, and these hospitals are advanced when compared to the facilities in the rural areas. The applicability of our results to the hospitals in the rural areas is unknown. Third, after building and testing the model, we found that only 47% of variance in adoption behavior could be explained. Even though this is higher than achieved by other studies, it is obvious that there are other factors that need to be considered.

## Competing interests

The authors declare that they have no competing interests.

## Author’s contributions

MS supervised the study, verified the data analysis and drafted the manuscript. PE collected the data and carried out the data analysis. NK and HN helped in the supervision of the study and editing the manuscript. All authors read and approved the final manuscript.

## Pre-publication history

The pre-publication history for this paper can be accessed here:

http://www.biomedcentral.com/1472-6947/12/142/prepub
